# Inertial Sensor-Based Sport Activity Advisory System Using Machine Learning Algorithms

**DOI:** 10.3390/s23031137

**Published:** 2023-01-19

**Authors:** Justyna Patalas-Maliszewska, Iwona Pajak, Pascal Krutz, Grzegorz Pajak, Matthias Rehm, Holger Schlegel, Martin Dix

**Affiliations:** 1 Institute of Mechanical Engineering, University of Zielona Góra, 65-417 Zielona Gora, Poland; 2 Institute for Machine Tools and Production Processes, Chemnitz University of Technology, 09126 Chemnitz, Germany

**Keywords:** mobile sensors (tags), anchors, fitness tracking, personal training, sport activities, sport activity advisory system, convolutional neural network (CNN), post-processing block (PPB), repetition counting

## Abstract

The aim of this study was to develop a physical activity advisory system supporting the correct implementation of sport exercises using inertial sensors and machine learning algorithms. Specifically, three mobile sensors (tags), six stationary anchors and a system-controlling server (gateway) were employed for 15 scenarios of the series of subsequent activities, namely squats, pull-ups and dips. The proposed solution consists of two modules: an activity recognition module (ARM) and a repetition-counting module (RCM). The former is responsible for extracting the series of subsequent activities (so-called scenario), and the latter determines the number of repetitions of a given activity in a single series. Data used in this study contained 488 three defined sport activity occurrences. Data processing was conducted to enhance performance, including an overlapping and non-overlapping window, raw and normalized data, a convolutional neural network (CNN) with an additional post-processing block (PPB) and repetition counting. The developed system achieved satisfactory accuracy: CNN + PPB: non-overlapping window and raw data, 0.88; non-overlapping window and normalized data, 0.78; overlapping window and raw data, 0.92; overlapping window and normalized data, 0.87. For repetition counting, the achieved accuracies were 0.93 and 0.97 within an error of ±1 and ±2 repetitions, respectively. The archived results indicate that the proposed system could be a helpful tool to support the correct implementation of sport exercises and could be successfully implemented in further work in the form of web application detecting the user’s sport activity.

## 1. Introduction

The effects of constantly changing lifestyles are leading to new challenges and potentials in daily life. Thus, the use of digital technologies is increasingly gaining importance in the fields of physiotherapy, fitness and health care in monitoring human activities in free training without additional supervision. The focus here on the research side is the usage of progressive sensor technologies based on multisensors in combination with machine learning (ML) tools for accurate sport activity recognition, as well as to improve computing power in order to build useful and practical tools [1].

For sport activity recording, the following wearables can be distinguished: chromatic smart glasses (for activity tracking for fitness purposes), smart watches (mostly integrated with headphones) and smart bands (for tracking the fitness of the user) [2]. Naturally, the accuracy of the measurements is a main issue; therefore, such devices can be complemented by new sensors. Inertial sensors have been widely applied in the study of human activity recognition (HAR), as well as in the context of sport activities. The authors of [3] analyzed the usage of inertial sensors in sport. It was found that further development is needed to support the so-called standardization of data collection and analysis procedures for physical activities [4] and to find a balance between the accuracy and practicability of the proposed solution. Moreover, although detecting the user’s activity and environment is beneficial, it is not yet sufficient to provide personalized advice for a specific type of sport.

Therefore, the goal of this research is to use inertia-based sensing analysis to provide sport activity recognition based on the example sport exercises of squats, pull-ups and dips and build a tool supporting training without a personal trainer through system feedback. It is known that applying artificial intelligence (AI) enables a system to support the automation of activities such as identification of correctly/incorrectly performed sport activities [5]. According to the statement that AI methods can help in training monitoring and training schedule optimization [6], in previous work [7], a critical analysis of the literature on this topic was performed from the point of view of convolutional neural network (CNNs) and of CNNs combined with classifiers for HAR applications in sports. In this work, we provide an overview of current solutions that support some feedback features for the correct performance of sports exercises with inertial sensors and CNNs (Table 1).

Table 1 presents examples of physical activity advisory systems found in the currently literature in order to enrich the discussion with a rapid overview of the main outcome of the presented systems with respect to the analyzed state of the art. In particular, Table 1 reports information on the related works about (a) the approach to advising on the realization of physical activity, (b) the adoption of wearables for activity recording, (c) the applied AI methods, and (d) the exploitation of the type of sport and the achieved accuracy. To the best of our knowledge and as already highlighted in the state-of-the-art analysis, no existing studies have developed a system supporting the correct implementation of sport exercises, namely squats, pull-ups and dips, using mobile sensors (tags) and stationary anchors in combination with a system-controlling server (gateway) and for data processing, i.e., a convolutional neural network (CNN) classifier with an additional post-processing block (PPB) and repetition counting. The authors of [31] suggested that research in the area of applications of AI in HAR in the context of the sport activities should focus on heterogeneous sensor fusion, combining expert knowledge with a combination of different AI methods to improve the accuracy of activity recognition systems. In previous research on sports activity detection using CNNs, only homogeneous signals with a certain type of activity were considered. According to the suggestion of the authors of [31] and to distinguish this work from previous studies, we applied CNN classifiers for the analysis of ongoing registered signals and for the classification of heterogeneous activity signals. In summary, the key contributions of this paper are as follows:

- Employment of three mobile sensors (tags), six stationary anchors and a system-controlling server (gateway) for 15 scenarios of the series of subsequent activities, namely squats, pull-ups and dips, as wearables for activity recording;
- Building a model supporting the identification of correctly performed activities of sport exercises, namely squats, pull-ups and dips, based on the data of 488 sport activities using a convolutional neural network (CNN) classifier with an additional post-processing block (PPB) and repetition counting;
- Design of an activity recognition module (ARM) and repetition-counting module (RCM) as the integral parts of the proposed advisory system;
- Achievement of satisfactory accuracy of the proposed system: CNN + PPB: non-overlapping window and raw data, 0.88; non-overlapping window and normalized data, 0.78; overlapping window and raw data, 0.92; overlapping window and normalized data, 0.87. For repetition counting, the achieved accuracies were 0.93 and 0.97 within an error of ±1 and ±2 repetitions, respectively, indicating that the proposed system is a helpful tool to support the correct implementation of sport exercises.

The remainder of this paper is organized as follows. In Section 2, we present an overview of our proposed advisory system for sport activities, namely squats, pull-ups and dips, as well as the process of data acquisition and analysis. In Section 3, we explores the experimental results and present the activity recognition module (ARM) and repetition-counting module (RCM) as the integral parts of the proposed advisory system. In Section 4, we discuss the achieved results, as well as directions for further works.

## 2. Materials and Methods

### 2.1. Overview of the System

**Figure 1:**
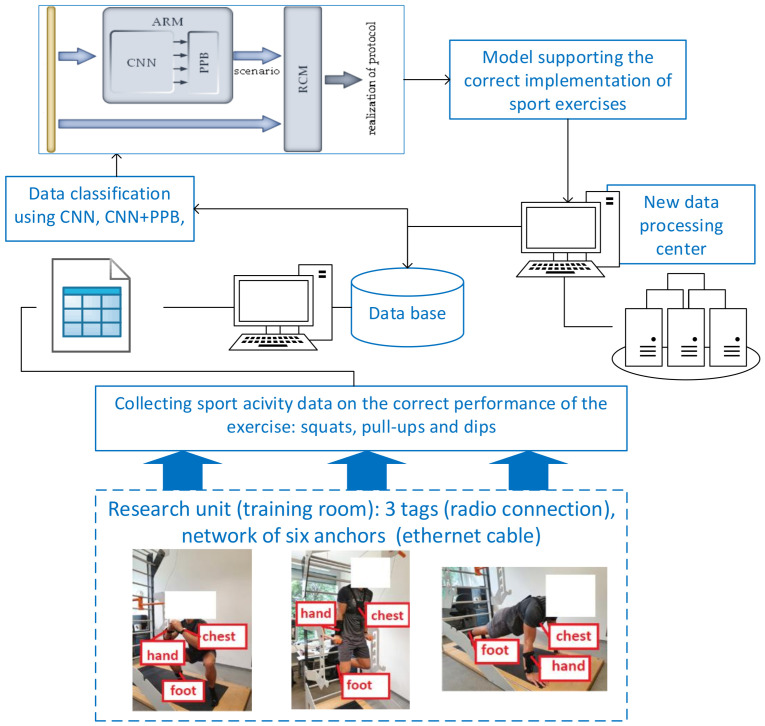
Schematic representation of the advisory system supporting the correct implementation of sport exercises, namely squats, pull-ups and dips.

The task of the advisory system considered in this paper is to supervise the correctness of the implementation of the training plan previously selected by the exercising person. The subjects perform the defined exercises (namely three activities: dips, pull-ups, and squats and breaks) in the developed research unit (Figure 1). The subjects wear three inertial measurement units (IMUs) on their chest, hand and foot. Therefore, the dataset includes three activities: dips and pull-ups with squats and breaks in between, with data collected in real time during exercises performed by eight participants. The recorded quantities include the axial size acceleration, rotation rate, magnetic field strength, the absolute position of the IMUs in the room, Euler angles, quaternions and pressure. A total of 20 measured quantities per IMU were received with a recording rate of 30 Hz. The maximum recording rate of the system is 100 Hz per tag, but performance problems occurred during data acquisition. The authors of [32] also showed that satisfactory classification of human activities can be achieved with sampling rates as low as 15 Hz. As in further work, the fusion of camera and sensor data is investigated, a 30 Hz recording rate was considered suitable. To develop a system supporting the identification of correctly performed activities of sport exercises, namely squats, pull-ups and dips, a validation module (VM) has to be designed. The proposed module consists of two main subsystems: an activity recognition module (ARM), which is responsible for extracting the series of subsequent sport activities, and a repetition-counting module (RCM), which determines the number of repetitions of a given activity in single series. Such an approach allows for the assessment of the activity compliance of the exercising person according to the previously established protocol (i.e., compliance with the scenario and the assumed number of repetitions). In our solution, the data are collected in real time, but in the presented solution, the posture of the exerciser is not detected. The purpose of the present research is to build a smart phone application acting as personal trainer, so this aspect was not taken into account in the advisory system presented in this paper.

Considering the originality of this work, an overview of the proposed advisory system supporting the correct implementation of sport exercises, namely squats, pull-ups and dips, is presented in Figure 1.

It is known that the methodology of modelling of the advisory system should be integrated with the use of scenario methods [33]. Therefore, our developed advisory system (Figure 1) includes: (1) data collection based on three mobile sensors (tags), six stationary anchors and a system-controlling server (gateway) during live sports exercise sessions (i.e., squats, pull-ups and dips); (2) 15 scenarios of the series of subsequent activities, namely squats, pull-ups and dips; (3) sport activity recognition using a convolutional neural network (CNN) with an additional post-processing block (PPB); (4) determination of the number of repetitions of a given activity; and (5) support for the correct implementation of sport exercises with the addition of new data.

### 2.2. Data Acquisition

A measurement system consisting of three mobile sensors (tags), six stationary anchors and a system-controlling server (gateway) developed by Pozyx was used to collect the data. The anchors are connected to the gateway via a patch cable, and the tags communicate via an ultra-wide band with the anchors distributed throughout the room. In addition to transmitting the inertial measurement variables, the system also determines the absolute position of the tags based on trilateration. This feature is particularly useful for monitoring several spatially distributed training stations. The data recorded by the tags are passed through the anchor lines to the gateway, which makes them available in the local network via MQTT. The recording and preprocessing of the MQTT data stream was achieved with MATLAB R2020b. Preprocessing consists of linear interpolation of the data when the transmission rate falls below 30 Hz and temporal synchronization of the three recorded sensor data streams. After preprocessing, the recorded data are available in an N × M matrix, where N corresponds to the number of samples and M represents the number of measured variables. During real-time data collection, the three exercises were performed by the subjects in several sequences, and one or more exercises could be performed within a sequence. The number of repetitions and the length of the pauses between individual sports exercises were carried out at the individual discretion of the test subjects. The subjects could also decide whether to perform all three exercises or only a selection thereof.

### 2.3. Dataset Analysis

The exercises were carried out in accordance with the protocols specifying the scenarios (i.e., sequences of activities) and the length of series of each activity (i.e., number of repetitions). It should be noted that the protocols were developed based on the activities performed sport by test persons. The exercises were carried out according to the sporting abilities of the test persons, and the order in which the exercises were completed was also decided by the test persons. The participants realized 78 protocols according to 15 scenarios with the number of activity repetitions ranging between 2 and 15. The used scenarios and the number of protocols are summarized in Table 2.

**Figure 2:**
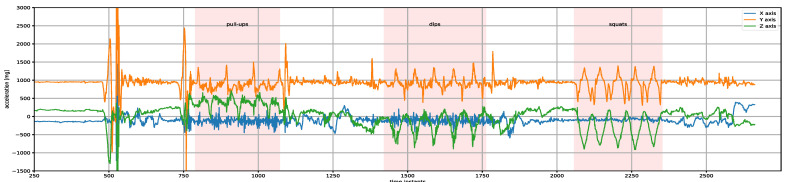
Raw acceleration signals from a sensor placed on the chest of a participant performing three pull-ups, five dips and five squats.

An example of raw acceleration signals from the sensor placed on chest of a participant registered while performing a protocol realized in accordance with the scenario comprising the pull-ups, five dips and five squats is presented in Figure 2.

**Figure 3:**
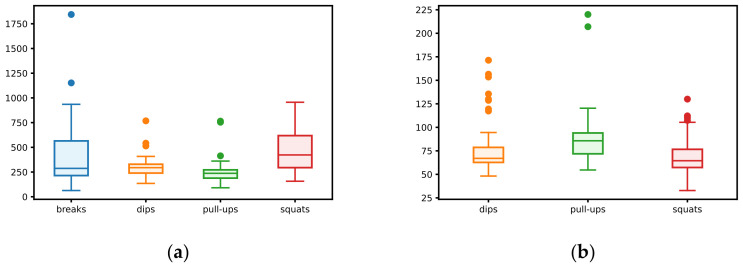
(**a**) Distribution of total time; (**b**) distribution of time per repetition of a given activity.

The raw signals collected during realizations of 78 protocols were split into parts containing one type of activity (break, dip, pull-up, squat). In further considerations, each of the parts obtained in this way is referred to as a single occurrence of the activity. The distribution of collected data is presented in Table 3 and Table 4 and Figure 3 (in all cases, time is expressed as the number of samples). The number of activity occurrences and the statistics of time duration in all scenarios within the protocols are shown in Table 3. The minimum and maximum number of repetitions and statistics of time duration of one activity repetition are presented in Table 4. In Figure 3, box-and-whisker plots show the distribution of total time and time per repetition based on the obtained dataset. Such analysis allows for determination of ranges of duration (expressed as the number of samples) for the occurrence of each activity, as well as the duration of one repetition. The parameters calculated in this way were used to tune the ARM and RCM modules of the advisory system described in detail in Section 3.

As shown in the Table 3 and Table 4, dataset is unbalanced because the distribution of activities is unequal, and the number of series of squats is approximately equal to the number of remaining activities (i.e., dips and pull-ups). A similar relationship can be observed in the duration of series (i.e., the number of registered samples); however, in this case, the differences are not so clear. The length of series (i.e., the number of repetitions) is similar for all activities and varies between 1 and 15. It takes the least amount of time to complete one squat, and the longest time is needed to complete one pull-up.

## 3. Results

**Figure 4:**
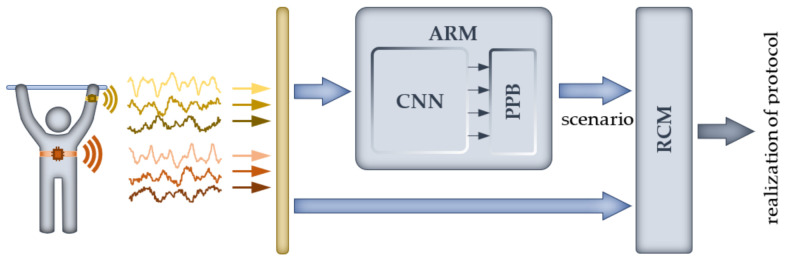
The scheme of the validation module.

As mentioned in the previous section, the proposed validation module (VM) that is a part of the designed advisory system (Figure 1) consists of two main subsystems. The first (ARM) is based on a CNN classifier and is responsible for the extraction of the scenario (the series of subsequent activities) performed by the exercising person. The second subsystem (RCM) uses information from the ARM and raw signals from sensors to determine the number of repetitions of a given activity. The scheme of the designed module is shown in Figure 4, and a detailed description of both subsystems is presented in the following subsections.

In order to design the ARM unit, a low-computational-cost CNN classifier based on our previous work is proposed. Owing to the nature of the task, in this work, the classifier was modified to process a live signal; moreover, it was supplemented with an additional post-processing block (PPB), enabling correct identification of the performed scenario. The second unit of the system (RCM) uses the known technique of finding peaks; however, the proposed approach to preprocessing of raw signals and tuning the parameters of the RCM block based on statistics of registered protocols is an original concept of the authors.

### 3.1. Activity Recognition Module

**Figure 5:**
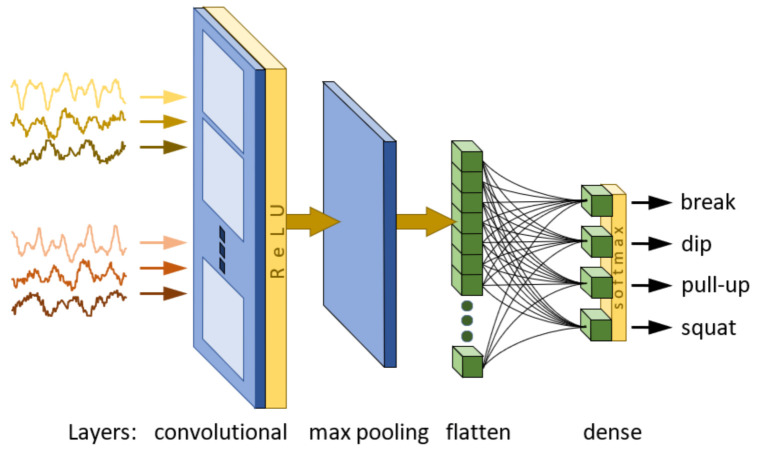
The general structure of the CNN classifier.

In order to design the ARM (Figure 4), an approach involving the use of a convolutional neural network (CNN) is proposed. The concept of this module is based on the results of our previous research on human activity recognition (HAR). In our previous work [7], a solution utilizing the technique known as image recognition [1,3] was proposed. The main goal of this work was to find a classifier structure demanding the lowest possible computational effort for the forward pass able to solve the HAR problem. A series of experiments involving a CNN with different hyperparameters allowed us to choose a classifier with one convolutional layer, five filters and a 7 × 1 kernel size. In [34], further research was conducted on decreasing computational effort by identification of significant sensor signals leading to input signal reduction. The studies focused on two issues: selecting the signals of greatest importance and sensor location. To evaluate the significance of the signals, an approach based on a comparison of the energy of signals transformed by a convolutional layer was used. In order to select sensor locations, the effectiveness of CNN classifiers for different sensor configurations was analyzed. As follows from the obtained results, the HAR problem can be satisfactorily solved using two acceleration sensors placed on the chest and the hand, which allowed for a reduction in the original set of 60 inputs to a set of only 6 signals. The results achieved in cited works constituted the basis for the studies presented in this paper. Finally, the classifier used in the experiments presented below is composed of one convolutional layer, five filters, a 7 × 1 kernel size, a ReLU activation function, a downsampling layer and a max pooling-type, flattened and dense output layer. The classifier input consists of 50 samples obtained from 6 acceleration signals from sensors placed on the chest and hand. The general structure of the employed CNN classifier is presented in Figure 5, and its parameters are listed in Table 5.

An important problem that had to be taken into account in the studies presented in this paper was the need to use a classifier for analysis of a live stream of data. In [7,34], the focus was on activity detection, and the input data of the CNN classifier contained only homogeneous signals covering one type of activity in both the training and testing phases. In contrast, in the presented work, the application of a CNN classifier for analysis of ongoing registered signals was considered, so the CNN had to also properly classify heterogenous activity signals. For this reason, two approaches for training the classifier were implemented and compared. In both cases, in order to prepare input data, the sliding-window method was used. In this technique, signals from sensors were sliced in the time domain to form 2D windows including 50 successive samples from 6 acceleration signals. In the first case, a non-overlapping window with homogenous signals presented in [34] was used; in the second case, a successive window overlap was used that can contain heterogenous activity signals. Additionally, in both cases, the classifier was trained and tested using raw and normalized signals.

The number of input data points (windows) used in training and testing phases is presented in Table 6. In the case of the overlapping window approach, the overlap of half the window size (25 samples) was used, and the number of windows including homogenous and heterogenous samples is presented separately. Based on assumption, each exercise scenario includes breaks between two activities, and according to Table 3, the duration the shortest break is equal to 63 samples, so heterogenous windows contain only single activities and breaks. For this reason, such data are treated as corresponding activities.

All experiments presented in this work were performed using algorithms implemented in Python 3.7.12. To create CNN classifiers with the structure shown in Figure 5, TensorFlow and Keras libraries in (version 2.7.0) were utilized. The code was run in the Google Colab environment with GPU support. In all experiments, datasets were split in a ratio of 70:15:15 between training, validation and test sets, respectively. The validation set with the early stopping approach was used to avoid classifier overtraining. In the case of the dataset created using the overlapping window method, the desired responses of the classifier were determined based on the percentage of samples corresponding to the activities registered within the window (i.e., for heterogenous data outputs less than 1). As a result of the training process based on the dataset created using the overlapping and non-overlapping window methods and raw and normalized data, four classifiers were obtained: C1 (non-overlapping window, raw data), C2 (non-overlapping window, normalized data), C3 (overlapping window, raw data) and C4 (overlapping window, normalized data). The accuracies of training and testing phases for these classifiers are presented in Table 7.

In all cases, the obtained results are satisfactory; however, the classifiers based on datasets created using a non-overlapping window achieve better accuracy in both the training and testing phases. Additionally, the accuracies obtained for normalized data are approximately equal (to the second decimal place) in contrast to raw data, which achieve significantly worse testing accuracy. The results presented in Table 7 allow for assessment of the quality of obtained classifiers, but they do not allow for the assessment of the effectiveness of classifiers operating as ARM, which should process the live stream of data.

**Figure 6:**
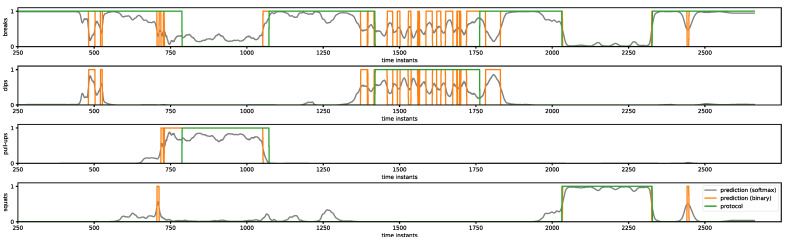
An exemplary result of live signal processing.

In real applications, the samples registered by the sensors arrive to ARM in real time and must be immediately processed by the classifier. Such a scenario can be interpreted as a sliding of the overlapping window in the time domain with a step equal to one sample. An exemplary result of live signal processing by classifier C4 to recognize the scenario in the protocol shown in Figure 2 is presented in Figure 6. The subsequent plots show output signals from the classifier corresponding to breaks, dips, pull-ups and squats. The gray line shows the raw output signal (probability of activity occurring), the orange line represents the output signal after conversion to binary classes and the green line describes the scenario used in the realized protocol. A simple conversion of the output signals to binary classes is insufficient because there are short periods of time (few subsequent samples) during which the activities are not correctly recognized and, as a consequence, converted to output signals containing peaks and gaps.

In order to eliminate the disadvantages mentioned above, the ARM was expanded with an additional post-processing block (PPB) for CNN output signals. As a result of experimentation, the two-stage algorithm was chosen. In the first step, each raw output signal of the CNN was smoothed with a moving average filter (MAF) according to dependency (1).

(1) $${\tilde{y}}_{i}=\frac{1}{w}\overset{w-1}{\underset{j=0}{\sum }}y_{i+j},$$

where $y_{i}$ and ${\tilde{y}}_{i}$ are the raw and filtered values, respectively, of the CNN output signal corresponding to the i-th sample, and w is the size of the window used by MAF. The output signal filtered in this way was converted to binary classes, and such a piecewise constant signal was processed in order to remove constant segments of insufficient length. For this reason, in the presented work, the eliminating peaks and gaps (EPG) algorithm was proposed according to dependency (2):

(2) $$Y_{i}=\left\{\begin{array}{cc} v & if\;\;\;\exists_{j}\;\;i\in P_{j}\\ \;1-v & otherwise \end{array}\right.,$$

where $Y_{i}$ is the output signal processed by EPG, v is equal to 1 or 0 for the EPG used to remove peaks or gaps, $P=\left\{P_{1},\ldots ,P_{j},\ldots ,P_{n}\right\}$ is a set of intervals with a constant signal (1 for removing peaks and 0 for removing gaps) with the lengths exceeding or threshold number of samples) for removing peaks and gaps, respectively.

**Figure 7:**
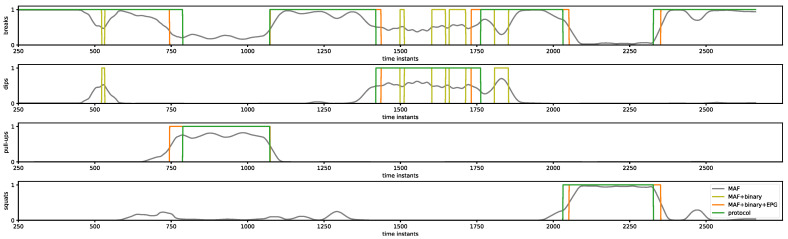
CNN output signal transformed by PPB filters.

In the presented work, parameters of filters described by dependencies (1) and (2) were assumed based on statistical measures of registered protocols shown in Table 3 as follows: w=50, $\delta_{1}=90$, $\delta_{0}=60$. To prepare a set of intervals (P) a function find_peaks from scipy.signal of Python module was used. The result of the CNN output signal shown in Figure 6 transformed by the PPB filters described above is presented in Figure 7.

As shown in Figure 7, after transformation by PPB using the MAF filter and binarization (olive line), most of the peaks and gaps in the output signal of the classifier shown in Figure 6 are removed. There are only a few peaks and gaps in the dip activity signal recognized incorrectly as breaks. After the EPG filter is added, the scenario is recognized correctly (orange line); however, slight shifts in time with respect to the protocol (green line) can be observed.

Finally, the accuracies of the considered classifiers and complete ARM unit (CNN + PPB) in activity recognition (Table 8) and scenario recognition (Table 9) were determined. The application of a post-processing block significantly improved the accuracy of both activity and scenario recognition. It is worth noting that the use of PPB significantly improved scenario recognition, which is especially important in the case of a system supporting the correct implementation of sport exercises. Based on the presented results, the ARM with a C3 classifier (overlapping window, raw data) was chosen as the component of the designed system.

### 3.2. Repetition-Counting Module

The second module of the designed system (Figure 4) has to calculate number of repetitions for each activity using information about activities provided by the ARM unit and raw signals registered by sensors. For this reason, in this study, the approach based on finding peaks in the original signals was used. Owing to the nature of the acquired signals (see Figure 2), their direct use for repetition counting led to overestimated results as a consequence of detecting false peaks. In order to eliminate this problem, preprocessing of original signals was performed.

**Figure 8:**
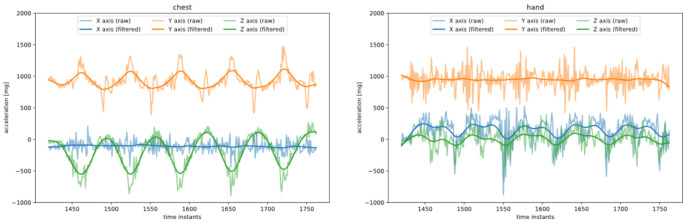
Acceleration signals from chest and hand sensors before and after filtering.

The preprocessing of raw signals should remove the noise resulting from sensor characteristics and accidental/unnecessary movements of the exercising person. To solve this problem, the application of a low-pass filter to attenuate signals with frequencies higher than the cutoff frequency was proposed. In this study, the Butterworth filter was chosen. The significant feature of this filter in eliminating false peaks is a maximally flat frequency response in the passband and near-zero response in the stopband. In this application, cutoff frequencies result from the time of performing single exercises. They were determined individually for each activity based on statistics presented in Table 4 and are listed in Table 10. The acceleration signals from chest and hand sensors registered during the performance of dips according to the protocol presented in Figure 2 before and after filtering are shown in Figure 8.

In the discussed system, each acceleration sensor provides three signals; nevertheless, as shown in Figure 2 and Figure 8, some signals do not contain information relevant to repetition counting (i.e., x-axis signal from the chest). To eliminate irrelevant signals, a series of experiments were carried out, and the chosen signals are presented in Table 10. Using data from sensors preprocessed and selected as described above, the number of repetitions of each activity was determined by counting the number of peaks of the signals. The parameters defining the minimum distances between the peaks required for correct performance of this operation were determined based on the minimum time needed to complete one repetition (Table 4) and are presented in last column of Table 10. Finally, the number of repetitions of each activity was determined as the average number of peaks across all previously chosen relevant signals.

To assess the effectiveness of the proposed system to supervise the correctness of the implementation of the training plan, a summary of obtained results is presented in Table 11 and Table 12. Table 11 contains the number of protocols, repetitions and miscounts grouped by activity. A low total number of miscounts was achieved, calculated as the total number of miscounts relative to the total number of repetitions (6%; 68/1105). The accuracies obtained in counting the repetition for each activity are shown in Table 12. Three types of accuracy were calculated: exact (ratio of correctly detected repetitions to the total number of repetitions), with errors of ±1 and ±2 repetitions. Accuracies within ±1 and ±2 equal 0.93 and 0.97, respectively, which are satisfactory, especially because they were determined based on scenario recognition performed by the ARM unit.

## 4. Discussion

In this work, an advisory system was developed to evaluate the correctness of the execution of sports activities (squats, pull-ups and dips). Each exercise was carried out according to the sporting abilities of the test persons, and the order in which the exercises were completed was also decided by the test persons. Based on the results, 15 scenarios of the series of subsequent activities were developed. Using three mobile sensors and six stationary anchors in combination with a system-controlling server (gateway), the data containing 488 sport activity occurrences were acquired. The proposed solution consists of two modules: an activity recognition module (ARM) and a repetition-counting module (RCM). The ARM, as a combination of a classifier and a post-processing block, showed very good performance in the recognition of three sports exercises with pauses in between. Squats were best-detected, which can be attributed to the predominant amount of data from squat intervals. Depending on the activity, classifiers C1 to C4 achieved different results. This should be investigated again with a more comprehensive dataset. The PPB block is an important component for the live recognition of sports exercises, especially when feedback functionalities have to be implemented.

Our results indicate that the developed advisory system can be treated as suitable tool to support the correct implementation of sport exercises. The developed system (CNN + PPB) achieved satisfactory accuracy:

- Non-overlapping window and raw data: 0.88;
- Non-overlapping window and normalized data: 0.78;
- Overlapping window and raw data: 0.92;
- Overlapping window and normalized data: 0.87.
- Repetition counting: 0.93 within an error of ±1 and 0.97 within an error of ±2.

The research results are comparable to another solution, and a higher recognition accuracy (0.97) was achieved when using repetition counting (see Table 1). The originality of this work is the combination of a convolutional neural network (CNN) with an additional post-processing block (PPB) and repetition counting. In addition, CNNs were used for the analysis of heterogeneous activity signals. The main limitation of the proposed system is that the protocols specifying the scenarios (i.e., sequences of activities) were developed based on the on the sport activities performed by test persons. This process of creating sport activity scenarios should be fully prepared by a sports trainer to guarantee the correctness of the exercises performed. In further work, the scenarios specifying the correctness of each sport activity will be formulated and added. The workflow and limitations described in this paper can also serve as a basis for further work in order to implement our advisory system in the form of a web application. This application will be a system acting as a personal trainer supervising the correctness of training in accordance with a previously prepared protocol (order, number and pace of exercises) using only sensors available in commonly used mobile devices (e.g., smart phones or smart watches).

## 5. Conclusions

In summary, the proposed advisory system that processes the data of 488 sports activities (squats, pull-ups and dips) collected from mobile sensors and stationary anchors provided through a system-controlling server (gateway) using a convolutional neural network (CNN) with an additional post-processing block (PPB) followed by repetition counting is a promising solution for the generation of a variety of conditions, providing the basis for a statement about the correctness of the performed sports exercise.

In a future work, sport activity scenarios prepared by a sports trainer will be applied, and experiments based on the new data will be conducted. Thus, the scenarios, new dataset and further research experiments on various neural networks to achieve better accuracy and to reduce the computational efforts would be especially beneficial for the development of a system in the form of, e.g., a web application available for users to supervise the performance of exercises without the need for the physical presence of a trainer.

## Figures and Tables

**Table 1:** A review of currently available solutions supporting the performance of sport exercises.

Physical Activity Advisory System	Applied Wearables for Activity Recording	Type of Sport	Applied AI	Achieved Accuracy	Ref.
Gym physical exercise recognition system	Single chest-mounted triaxial accelerometer	Six muscle groups, gym exercise	Long short-term memory (LSTM) neural networks	Non-overlapping dataset: 0.57–0.81; overlapping dataset: 0.74–0.91	[8]
System for weight training	An inertial measurement unit (IMU), an accelerometer and three force sensors	Weightlifting	Nearest neighbor (KNN), decision tree (DT), random forest (RF) and repetition counting	KNN: 0.97; DT: 0.99; SVM: 0.98; RF: 100; repetition counting: 0.96	[9]
Recognizing gym workouts	A micro-watt-level power consumption human body capacitance based sensor	Seven gym workouts	Repetition counting	0.91	[10]
Fitness activity recognition	Microphone integrated into a smartphone	Body weight exercises, bicycles, toe touches and squats	Support vector machines (SVMs) and convolutional neural networks (CNNs)	0.88 for bicycles, 0.97 for toe touches and 0.9 for squats	[11]
Exercises in CrossFit recognition	Smart watches in communication with each other over Bluetooth low-energy (BLE)	10 CrossFit exercises	Classifiers of a series of 2D convolutions and two fully connected layers, as well as repetition counting	0.99; repetition counting: 0.91	[12]
Shoulder physiotherapy exercise recognition	Six-axis inertial sensor	Seven shoulder exercises	K-nearest neighbor (k-NN), random forest (RF), support vector machine (SVM) and convolutional recurrent neural network (CRNN)	Classification algorithms: 0.94; CRNN algorithm: 0.99	[13]
Static and dynamic activity recognition	Two radio devices (waist- and ankle-worn) based on measurements of variations in the received signal strength	Static (standing, sitting and lying) and dynamic activities (walking, running and dancing)	K-nearest neighbor (k-NN), support vector machine (SVM) and a combination thereof	0.99	[14]
Daily activity recognition	Recorded with smart watch and smart phone	Six non-hand-oriented activities	Convolutional neural networks (CNNs), long short-term memory (LSTM) and combinations thereof	Hybrid LSTM: 0.99	[15]
Magnetic-induction-based human activity recognition	Wireless system based on magnetic induction	Walking and knocking	Deep recurrent neural networks (DRNNs)	0.88 (synthetically generated motion dataset)	[16]
Sport activity recognition	IMU attached to the chest	Walking, jogging, sprinting and jumping	Decision trees (DT), discriminant analysis, support vector machine (SVM) and k-nearest neighbor (k-NN)	Cubic SVM: 0.91	[17]
Badminton activity recognition	Accelerometer/gyroscope sensors attached to the wrist, upper arm and racket grip	Seven different strokes and two movement types	Convolutional neural networks (CNNs) and Deep CNNs	CNN: 0.99 (with accelerometer and gyroscope data)	[18]
Beach volleyball activity recognition	Three-axis acceleration sensor worn on the dominant hand	Ten action classes in beach volleyball	Deep convolutional neural networks (DCNNs)	DCNN: 0.83	[19]
Motion activity recognition based on Wi-Fi signal analysis	Two laptops equipped with three omnidirectional antennae and analysis of channel-state information	Nine motion activities (e.g., walking, paper toss and hand clap)	Decision tree (DT), convolutional neural networks (CNNs) and long short-term memory (LSTM)	DT: 0.94	[20]
Activity recognition in real time	Textile-based capacitive sensors implemented as knee braces	Walking, standing, running and squatting	Support vector machine (SVM), decision tree (DT), k-nearest neighbor (k-NN) and random forest (RF)	RF: 0.83	[21]
Table tennis activity recognition	Six-axis accelerometer/gyroscope attached to the arm	Five typical table tennis strokes	Support vector machine (SVM) and k-nearest neighbor (k-NN)	SVM: 0.96	[22]
Activity recognition with additional photoplethysmographic signals	Accelerometer and PPG sensor data	Walking, running, cycling (with high and low resistance)	Bayesian classifier	0.78	[23]
Activity recognition	Heart-rate-monitoring wrist band equipped with a triaxial accelerometer	Home-specific activities (sitting, standing, household activities and stationary cycling)	Support vector machine (SVM) and random forest (RF)	SVM: 0.85; RF: 0.89	[24]
Field hockey activity recognition	Two IMUs worn on the chest and waist	Six field hockey activities	Cubic support vector machine (SVM)	0.91	[25]
Fitness activity recognition	Thermal vision sensor	Push-ups, sit-ups, jumping jacks, squats and planks	Convolutional neural networks (CNNs) for feature extraction and long short-term memory (LSTM) for final classification	0.98	[26]
Walking activity recognition	A heterogeneous sensor system: leg-worn IMU and finger-tip-based pulse sensors	Walking activity and leg-swing activities	Deep convolutional neural network (DCNN)	0.97	[27]
Recognition of the physical activities of children	Three-axis accelerometer modules around the waist	Slow/fast walking, slow/fast running, walking up/down stairs, jumping rope, standing up and sitting down	Convolutional neural network (CNN)	0.81	[28]
Hand-oriented activity recognition	Triaxial accelerometer data and triaxial gyroscope	Jogging and walking (public benchmark dataset called WISDM)	Convolutional neural network (CNN) and long short-term memory (LSTM)	0.96	[29]
Daily life activity recognition	Two-axis smart phone accelerometer sensor	Jogging and walking (public benchmark dataset called WISDM)	Multilayer perceptron (MLP) classifier	0.93	[30]

**Table 2:** The used scenarios and the number of protocols.

Scenario	Number of Protocols
dips	1
pull-ups	2
squats	7
dips, squats	3
pull-ups, squats	1
squats, dips	1
squats, squats	5
dips, pull-ups, squats	5
dips, squats, pull-ups	4
pull-ups, dips, squats	28
pull-ups, squats, dips	1
squats, dips, pull-ups	4
squats, pull-ups, dips	2
squats, squats, squats	13
pull-ups, dips, squats, squats	1

**Table 3:** The number of activity occurrences and the statistics of duration.

Activity	Number of Occurrences	Duration in Number of Samples		
		Min	Avg	Max
breaks	283	63	382	1844
dips	50	135	297	768
pull-ups	48	91	249	765
squats	107	157	474	956
Total	488	63	380	1844

**Table 4:** The number of repetitions and the statistics of the duration of one activity repetition.

Activity	Repetitions		Duration in Number of Samples		
	Min	Max	Min	Avg	Max
dips	2	12	48	79	171
pull-ups	1	14	55	90	220
squats	2	15	33	68	130
Total	1	15	33	76	220

**Table 5:** Parameters of the CNN classifier.

Layer	Layer Type	Output Shape	Number of Parameters
1.	Convolution (5 filters, 7 × 1 kernel, ReLU)	(44, 6, 5)	5 × (7 + 1)
2.	Max pooling	(22, 6, 5)	0
3.	Flatten	(660)	0
4.	Dense (softmax)	(4)	4 × (660 + 1)
Total parameters			2684

**Table 6:** The number of input data points used in the training and testing phases for both approaches.

Activity	Non-Overlapping Window	Overlapping Window	
		Homogenous Data	Heterogenous Data
breaks	2024	3812	-
dips	273	493	192
pull-ups	215	384	189
squats	956	1810	423
Total	3468	7303	

**Table 7:** The accuracies of training and testing phases for obtained classifiers.

Phase	Non-Overlapping Window		Overlapping Window	
	Raw Data Classifier C1	Normalized Data Classifier C2	Raw Data Classifier C3	Normalized Data Classifier C4
training	0.98	0.94	0.95	0.92
testing	0.93	0.94	0.90	0.92

**Table 8:** The accuracies for single and all activities.

Activity	CNN Classifiers				ARM (CNN + PPB)			
	C1	C2	C3	C4	C1	C2	C3	C4
dips	0.74	0.63	0.79	0.72	0.91	0.67	0.97	0.8
pull-ups	0.77	0.87	0.78	0.9	0.91	0.93	0.91	0.95
squats	0.9	0.93	0.86	0.92	0.95	0.97	0.94	0.97
Total	0.85	0.86	0.83	0.88	0.94	0.91	0.94	0.93

**Table 9:** The accuracies for scenario recognition.

CNN Classifiers				ARM (CNN + PPB)			
C1	C2	C3	C4	C1	C2	C3	C4
0.00	0.13	0.00	0.21	0.88	0.78	0.92	0.87

**Table 10:** RCM parameters.

Activity	Signals						Butterworth Filter		Find Peaks
	Chest			Hand					
	X Axis	Y Axis	Z Axis	X Axis	Y Axis	Z Axis	Order	$W_{n}$ *	Distance
dips	□	□	☑	☑	□	☑	2	0.05	40
pull-ups	□	☑	☑	□	□	☑	2	0.04	50
squats	□	□	☑	□	□	□	2	0.06	30

* $W_{n}=f_{c}/\left(0.5f_{s}\right)$—normalized cutoff frequency, $f_{c}$—cutoff frequency, $f_{s}$—sampling frequency, ☑ given signal is used in RCM module, □ given signal is not used in RCM module.

**Table 11:** Repetition count results.

Activity	No. of Protocols	Total Repetition Count	Total Miscount
dips	51	202	20
pull-ups	49	144	19
squats	75	759	29
Total	175	1105	68

**Table 12:** Accuracies of repetition counting.

Activity	Exact	within ±1	within ±2
dips	0.78	0.90	0.94
pull-ups	0.67	0.96	0.98
squats	0.77	0.92	0.97
Total	0.75	0.93	0.97

## Data Availability

Not applicable.
